# Applying population health approaches to improve safe anticoagulant use in the outpatient setting: the DOAC Dashboard multi-cohort implementation evaluation study protocol

**DOI:** 10.1186/s13012-020-01044-5

**Published:** 2020-09-21

**Authors:** Geoffrey D. Barnes, Emily Sippola, Michael Dorsch, Joshua Errickson, Michael Lanham, Arthur Allen, Patrick Spoutz, Anne E. Sales, Jeremy Sussman

**Affiliations:** 1 University of Michigan Frankel Cardiovascular Center and Institute for Healthcare Policy and Innovation, 2800 Plymouth Rd, B14 G214, Ann Arbor, MI 48109-2800 USA; 2 University of Michigan Center for Bioethics and Social Science in Medicine, Ann Arbor, USA; 3 University of Michigan School of Pharmacy and Institute for Healthcare Policy and Innovation, Ann Arbor, USA; 4 University of Michigan Center for Statistical Consultation and Research, Ann Arbor, USA; 5 University of Michigan Department of Learning Health Sciences, Ann Arbor, USA; 6 VA Salt Lake City Health Care System, Salt Lake City, USA; 7 Veterans Health Affairs VISN 20 Pharmacy Benefits Management, Vancouver, USA; 8 University of Michigan Department of Learning Health Sciences, Institute for Healthcare Policy and Innovation, and Ann Arbor Veterans Health Affairs Center for Clinical Management and Research, Ann Arbor, USA; 9 University of Michigan Department of Internal Medicine, Institute for Healthcare Policy and Innovation, and Ann Arbor Veterans Health Affairs Center for Clinical Management and Research, Ann Arbor, USA

**Keywords:** Anticoagulant, Pharmacists, Implementation science, Electronic health records, Medication errors

## Abstract

**Background:**

Use of direct oral anticoagulants (DOAC) is rapidly growing for treatment of atrial fibrillation and venous thromboembolism. However, incorrect dosing of these medications is common and puts patients at risk of adverse drug events. One way to improve safe prescribing is the use of population health tools, including interactive dashboards built into the electronic health record (EHR). As such tools become more common, exploring ways to understand which aspects are effective in specific settings and how to effectively adapt and implement in existing anticoagulation clinics across different health systems is vital.

**Methods:**

This three-phase project will evaluate a current nation-wide implementation effort of the DOAC Dashboard in the Veterans Health Administration (VHA) using both quantitative and qualitative methods. Informed by this evaluation, the DOAC Dashboard will be implemented in four new health systems using an implementation strategy derived from the VHA experience and interviews with providers in those new health systems. Quantitative evaluation of the VHA and non-VHA implementation will follow the Reach, Effectiveness, Adoption, Implementation, Maintenance (RE-AIM) framework. Qualitative interviews with stakeholders will be analyzed using the Consolidated Framework for Implementation Research and Technology Acceptance Models to identify key determinants of implementation success.

**Discussion:**

This study will (1) evaluate the implementation of an EHR-based population health tool for medication management within a large, nation-wide, highly integrated health system; (2) guide the adoption in a set of four different health systems; and (3) evaluation that multi-center implementation effort. These findings will help to inform future EHR-based implementation efforts in a wide variety of health care settings.

Contributions to the literature

- This study will evaluate the implementation of a multi-center electronic health record-based population health tool for anticoagulant medication management.
- Informed by a qualitative analysis in a highly integrated health system, an implementation plan will be developed and deployed in a different multi-center health system collaborative.
- This study will help explore the impact of population health tools for safe anticoagulant medication management in a variety of different clinical contexts

## Background

For more than five decades, warfarin was the only available oral anticoagulant in the USA and much of the world. Since 2010, four new direct oral anticoagulants (DOACs) have been introduced into the market. All are approved by the US Food and Drug Administration (FDA) for the prevention of stroke in non-valvular atrial fibrillation (AF) and to prevent or treat venous thromboembolism (VTE). The DOACs require a different management approach than warfarin [1]. Unlike warfarin, (1) no reliable and widely available laboratory tests exist to monitor drug levels, (2) each drug is excreted in part through the kidneys, (3) drug-drug interactions impact DOAC dosing and safe use differently when compared to warfarin, and (4) dosing of DOAC medications is unique for each indication (AF, VTE, and coronary/peripheral artery disease). In fact, multiple studies have identified that as many as 1 in 7 patients have inappropriate DOAC prescriptions [2–5]. When DOACs are used inappropriately (over-dosing, under-dosing, and dosing based on the wrong indication), patients are at markedly increased risk for costly and potentially deadly bleeding and thrombotic/stroke complications [3].

Many warfarin-treated patients are managed by anticoagulation clinics due to the drug’s complex pharmacokinetics and the need for frequent laboratory-guided dose adjustment [6]. These clinics are staffed by pharmacists and nurses with expertise in thrombotic disorders and anticoagulation, have shown to improve the quality of anticoagulation care, and are associated patient outcomes over standard of care [7, 8]. The US Veterans Health Administration (VHA) has been a national leader in the use of anticoagulation clinics. Anticoagulation clinics are also common outside the VHA and may improve the safe use of warfarin [8–12].

The VHA is the largest vertically and horizontally integrated health systems in the world [13]. Serving over 9 million veterans in the USA, it offers services through 170 medical centers throughout the 50 states, Puerto Rico, Guam, Philippines, American Samoa, and Virgin Islands. These medical centers have over 1000 affiliated outpatient clinics, over 100 nursing homes, and other health facilities focused on care for homeless veterans, behavioral health, and substance use disorders.

Given the robust VHA network of anticoagulation clinics managing warfarin-treated patients, we previously suggested that all DOAC-treated patients should be managed similarly [14]. However, without the burden of frequent laboratory tests or dose adjustment, most prescribers do not routinely refer DOAC-treated patients to anticoagulation clinics [6]. Doing so would likely require prohibitive resources at current staffing levels. Yet half of all anticoagulation clinics provide some DOAC support, and these clinics are looking for tools to broaden their reach and impact while improving efficiency [6, 15]. Expansion of anticoagulation clinics to identify and support the care of DOAC-treated patients requires a novel approach since they are not referred as commonly as warfarin-treated patients.

Provider education is unlikely to solve this significant quality gap because of the large number of uncommon but important risks. These include (1) a variety of drug-drug interactions to both common and rare drugs; (2) different adjustments for renal function for each different drug, all of which use the Cockcroft-Gault creatinine clearance [16] rather than the estimated glomerular filtration rate equation [17]; and (3) the nuanced differences in dosing for AF and VTE for each DOAC medication. While some patients are intentionally under-dosed due to high-risk for bleeding, these errors are often unintentional and can lead to both under- and over-dosing of DOAC medications. In addition, many providers see relatively few patients taking these medications, so that the medication risks and dosing nuances are not necessarily remembered easily and addressed.

EHR alerts and dosing guidance might encourage appropriate dosing. However, alert fatigue has led many health systems to either deactivate electronic health record (EHR) drug alerts or display only the highest-risk alerts [18, 19]. Additionally, many of the DOAC dosing issues may develop after the initial prescription (e.g., progressive renal function decline, failure to change VTE lead-in dosing for apixaban or rivaroxaban after 7 or 21 days, respectively) [20, 21]. Therefore, an EHR-based alert targeted at a prescribing provider is unlikely to be highly effective.

Population health tools, such as dashboards, leverage the power of the EHR to identify patients within a large population (e.g., an entire health system) who match certain criteria (e.g., being prescribed an oral anticoagulant) and then screen for any potential red flags (e.g., inappropriate dosing for a given renal function). While not able to address all components of high-quality medication use, these tools address one critical component (appropriate dosing) in an efficient manner [22]. Such tools are currently being used for population-based management of patients with diabetes [23]. However, integration into clinical work flow and responsiveness to user feedback remain major obstacles for implementation and broad dissemination. It is also unclear how implementation success will differ between fully integrated health systems, such as VHA which is publicly funded through a capitated system, and health systems with more insurance-based funding models that pay using fee-for-service models.

The DOAC Dashboard study will evaluate and compare the implementation of a DOAC population health tool within the nation-wide VHA health system as well as four diverse health systems participating in the Michigan Anticoagulation Quality Improvement Initiative (MAQI^2^). Quantitative implementation evaluation will use nation-wide data from VHA and federal resources, where the DOAC Dashboard was implemented beginning in 2016, as well as data from the MAQI^2^ collaborative, where the DOAC Dashboard was implemented starting in 2020. Qualitative evaluation will compare determinants of implementation success in fully integrated VHA facilities with less integrated health systems participating in the MAQI^2^ collaborative.

## Methods

### Specific aims

1. Evaluate the effectiveness, implementation successes, and limitations of the DOAC Dashboard for safe DOAC prescribing within the national VHA system. To do this, we will measure the reach, effectiveness, adoption, implementation, and maintenance (RE-AIM) of the VHA DOAC Dashboard using both patient-level and provider-/facility-level measures. By linking data from the VHA Clinical Data Warehouse with DOAC Dashboard utilization data from VA Pharmacy Benefits Management, we will identify high- and low-user centers, individual patient and prescriber characteristics, DOAC dosing errors, and DOAC adverse event rates (bleeding and thrombotic events).
2. Use a systematic approach to design an implementation plan for DOAC Dashboard use outside of VHA. Informed by stakeholder qualitative interviews at VHA and non-VHA sites, we will identify key determinants of implementation success. These determinants will be paired with targeted strategies for use during the MAQI^2^ implementation.
3. Evaluate the implementation of a DOAC Dashboard for safe DOAC management within four unique and diverse health systems. Informed by the design and implementation of the VHA DOAC Dashboard, we will adapt and implement a DOAC Dashboard at four health systems participating in MAQI^2^. Using the existing MAQI^2^ registry, we will evaluate this implementation using the RE-AIM framework. We will also perform qualitative interviews at each site to assess acceptability of this care model.

### Design

The DOAC Dashboard study was reviewed and approved or deemed “not regulated” by the University of Michigan (HUM00162234 and HUM0021922) and VA Ann Arbor Institutional Review Boards (IRB-2018-1101). The study is conducted in two settings. The MAQI^2^ collaborative of health systems in Michigan include non-governmental urban and suburban hospitals with and without academic affiliations. The MAQI^2^ collaborative is funded by Blue Cross Blue Shield of Michigan, a health insurance provider, to improve the quality of anticoagulation care in the state of Michigan. Participating hospitals enter data into a shared data registry and collaboratively work on quality improvement efforts. To date, data on more than 3800 patients taking DOACs with more than 8800 6-month follow-ups have been entered into the MAQI^2^ registry. The four participating sites in this project all enter patients taking DOAC medications into the MAQI^2^ registry and all use the same EHR vender (Epic®). Quantitative and qualitative evaluations will be performed in both settings, guided by the RE-AIM, CFIR, and TAM implementation models/frameworks. Any significant protocol modifications will be communicated in journal publications after review and approval by the study leadership team. Final quantitative datasets will remain under the supervision of the VHA and MAQI^2^ research teams.

#### Phase I—Evaluate the VHA DOAC Dashboard implementation

Evaluation of the current VHA DOAC Dashboard (sample screen shots in online appendix) will follow the RE-AIM framework (Table 1) [24].

**Table 1:** RE-AIM evaluation of DOAC Dashboard implementation

RE-AIM dimension	Outcome measure	How assessed	Data source
Reach	Number of DOAC-treated patients cared for by sites using the DOAC Dashboard	Calculate the number (and percent) of active DOAC-treated patients at sites with ongoing DOAC Dashboard use (at least weekly) on a month-by-month basis Broken down by implementation phase: Early (8/2016-3/2017), Mid (4/2017-12/2017), Late (1/2018-12/2018)	VHA PBM database of DOAC Dashboard use
Effectiveness	(1) Inappropriate DOAC use	Dependent variable: appropriate vs. inappropriate DOAC prescription (FDA prescribing instructions) assessed for each individual patient Primary independent variable: frequency of DOAC Dashboard use normalized for site-level number of DOAC prescriptions Important covariates: site, date, patient characteristics (e.g., demographics, comorbidities), site characteristics (e.g., urban/suburban/rural, academic, percent Black patients)	VHA CDW and PBM database of DOAC Dashboard use
	(2) Adverse events	Dependent variable(s): ICD codes for stroke, venous thromboembolism, and bleeding (see online appendix) as well as all-cause death Independent variables: same as for primary analysis	
Adoption	Site-level use of DOAC Dashboard	Numerator: number of VHA sites with DOAC Dashboard use (any, weekly, daily) Denominator: total number of VHA sites	VHA PBM database of DOAC Dashboard use
Implementation	Percent of DOAC Dashboard alerts fixed within 7 days	Calculate the percent of new prescribing errors (DOAC Dashboard alerts) that are resolved within 7 days stratified by site level of DOAC Dashboard use	VHA CDW and PBM database of DOAC Dashboard use
Maintenance	Sustainment of DOAC Dashboard use	Early adopters—plot the frequency of use for sites that began DOAC Dashboard use during the early phase (8/2016-3/2017) Broad adoption—plot overall site-level DOAC Dashboard use following broad adoption in early 2018	VHA PBM database of DOAC Dashboard use

*VHA* Veteran’s Health Affairs, *DOAC* direct oral anticoagulant, *PBM* Pharmacy Benefits Manager, *FDA* Food and Drug Administration, *CDW* corporate data warehouse

Reach will be calculated as the number (and percent) of patients taking DOAC medications who are managed at sites with regular (at least weekly) DOAC Dashboard use. This will be calculated month-by-month during the entire study period as well as during the 3 implementation phases (defined in Table 1). VHA sites with any tool use as well as number of sites based on level of DOAC Dashboard use. Use will be calculated as the number of days each month with one or more DOAC Dashboard log in. Characteristics of the DOAC prescribers and anticoagulation clinics will be assessed for DOAC Dashboard user and non-user sites in each of three implementation phases (defined in Table 1).

The primary outcome of our evaluation of the effectiveness of the DOAC Dashboard will be inappropriate DOAC prescribing. The core secondary outcomes will be DOAC-related adverse events. Our primary predictor will be the degree of DOAC Dashboard use (number of days with any DOAC Dashboard use in the month of DOAC prescription). All analyses will start in 2014 (2 years before the introduction of the DOAC Dashboard) and continue through at least the end of 2019.

We will use a logistic mixed effects regression model to examine whether there is a decline in the rate of inappropriate DOAC prescribing at sites with higher use of the dashboard. The primary outcome variable will be inappropriate DOAC use. The main predictor of interest will be the number of days with any DOAC Dashboard log in recordings in the month of each prescription normalized for the number of DOAC patients at that site. This provides a reasonable approximation of DOAC Dashboard use. We will explore non-linear relationships between DOAC Dashboard use and the inappropriate DOAC use. We will adjust for clinical and socioeconomic characteristics, including patient age, sex, race, indication for DOAC use, creatinine clearance, risk of strokes with the CHA_2_DS_2_-VASc score [25], risk of bleeding using the HAS-BLED score [26], and comorbidity using the Charlson score with Deyo modification [27], date, and site-level characteristics, including percent of patients who are rural and percent of patients who are Black. We will account for clustering using VHA facility as a random effect.

For the secondary outcome measures, we will individually measure bleeding, VTE, stroke events, and all-cause death using Poisson or negative binomial mixed effects regression models. We will also explore a composite endpoint of bleeding, VTE, stroke, and all-cause death in a similar model. In addition to the predictor variables listed above, we will also include a variable to account for length of DOAC treatment at the patient level, as this likely would influence a patient’s risk of adverse events. Details about adverse event ascertainment can be found in the online appendix.

In sensitivity analyses, we will conduct an interrupted time series analysis to explore how different levels of DOAC Dashboard use associate with appropriate DOAC prescribing. We will use three different time points in these sensitivity analysis: (1) date when each site first used the DOAC Dashboard, (2) date when each site first had at least weekly DOAC Dashboard use (averaged over a 4 week period), and (3) date when each site first had daily DOAC Dashboard use (averaged over a 5-consecutive-day period). In this approach, the number of sites achieving each of the time points may vary, as some sites may not achieve daily Dashboard use during the study period.

#### Phase II—Systematic approach to implementation planning

Determinants of implementation success will be evaluated in two different settings. First, we will assess realized determinants of implementation success in the national VHA health system. This will be done by conducting semi-structured interviews with key DOAC Dashboard stakeholders (primarily pharmacists, but also physicians, nurses, managers) at different VHA centers within each use category (low/no, moderate, high). We will also identify centers with notable changes in use (e.g., initially high but then low/no use) to explore what determinants of implementation success impacted change in DOAC Dashboard use. Draft interview guide is included in the online supplemental appendix.

Second, we will assess anticipated determinants of implementation success at four non-VHA health centers participating in the MAQI^2^ consortium. These semi-structured interviews will include the same stakeholders as in the VHA interviews. However, these will occur before any MAQI^2^ site has begun to implement the DOAC Dashboard. An orientation to the DOAC Dashboard using screen shots with blinded patient data will be shown to the interviewees.

Finally, after implementation of the DOAC Dashboard has been completed at the MAQI^2^ site, we will re-interview stakeholders to assess their experienced determinants of implementation success. The same stakeholders will be approached, when available. New stakeholders will also be approached as appropriate.

Potential interviewees will be identified both from institutional records of who manages anticoagulation at the relevant health systems as well as through recommendations from previously interviewed stakeholders. New semi-structured interviews will be conducted until either thematic saturation is reached or all potential stakeholders have been approached. The latter is more likely to occur within the MAQI^2^ sites given the smaller number of potential sites (4) and stakeholders than are present within the VHA system.

After transcribing and anonymizing each interview, we will undertake a template analysis, a form of thematic analysis which uses pre-determined codes from existing frameworks (e.g., CFIR, TAM) and then refines those codes as transcripts are coded and analyzed [28]. New themes that emerge from the transcripts will be included with the pre-existing themes included from CFIR and TAM. Emergent themes that do not fit well into either CFIR or TAM will be explored using other implementation determinants frameworks (e.g., Theoretical Domains Framework [29]), or potentially as new constructs.

Themes identified from both the VHA interviews as well as the MAQI^2^ initial interviews will be paired with relevant implementation strategies following a behavior change technique [30, 31]. We will leverage the existing quality collaborative infrastructure, including quarterly meetings, to share key implementation resources for the DOAC Dashboard.

#### Phase III—Implement and evaluate the DOAC Dashboard in MAQI^2^ health systems

An adaptation of the VHA DOAC Dashboard will be designed within the Epic EHR system. Working iteratively with anticoagulation clinic staff, the MAQI^2^ DOAC Dashboard will identify patients prescribed DOAC medications and who have a primary care provider, cardiologist, hematologist, or vascular surgeon that they follow with in the ambulatory setting. The dashboard will then identify potential medication or dosing errors based on indication, drug-drug interaction, or renal function. Implementation support, including EHR support specific to the DOAC Dashboard, will be provided by the research team. Each participating MAQI^2^ system will determine how best to operationalize dashboard implementation and utilization. The research team will interview anticoagulation clinic managers about specific implementation strategies employed and operational details about use of the Dashboard. Ongoing data collection through the MAQI^2^ consortium will be utilized to assess implementation success [32].

Similar to the phase I evaluation of the VHA DOAC Dashboard, evaluation of the MAQI^2^ DOAC Dashboard will also follow the RE-AIM framework (Table 2) [24].

**Table 2:** RE-AIM evaluation of MAQI^2^ DOAC Dashboard implementation

RE-AIM dimension	Outcome measure	How assessed	Data source
Reach	The percent of patients with inappropriate DOAC use where the anticoagulation clinic contacted the prescribing provider or patient	Numerator: number of patients where prescriber or patient was contacted about inappropriate DOAC prescribing Denominator: total number of patients with DOAC Dashboard alerts for inappropriate medication prescribing	MAQI^2^ DOAC Database
Effectiveness	Inappropriate DOAC use	Interrupted time series analysis Numerator: Number of inappropriate DOAC prescriptions Denominator: Total number of patients on DOAC	MAQI^2^ DOAC Database
Adoption	Implementation strategies used	Qualitative interviews with anticoagulation clinic managers	Interviews
Implementation	Time between a new DOAC Dashboard flag and a clinical change	Time in days between the occurrence of a new DOAC Dashboard flag and documented change in DOAC medication	MAQI^2^ DOAC Database
Maintenance	Sustained frequency of Dashboard use	Number of days per month with at least one Dashboard access by any staff member over a 12-month period	EHR report

*MAQI*^*2*^ Michigan Anticoagulation Quality Improvement Initiative, *DOAC* direct oral anticoagulant, *EHR* electronic health record

Unlike for phase I in which we have access to a national dataset of patients prescribed DOAC mediations, the MAQI^2^ will have fewer patients and therefore fewer DOAC-related complications (e.g., bleeding, thrombosis). Therefore, the effectiveness outcome will focus on appropriate prescribing rather than adverse event rates, which can be extrapolated from VHA data and other larger data sources. With alpha level of 0.05, we will have > 80% power to detect > 2.5% absolute reduction in the inappropriate DOAC use after the introduction of DOAC Dashboard from a MAQI^2^ baseline of 10% inappropriate use prior to DOAC Dashboard implementation.

### Plans for dissemination

The study team anticipate disseminating findings through presentations at national and/or international scientific meetings and in peer reviewed manuscripts. Manuscripts will be authored by study team members without the use of professional writers. All authors will meet the appropriate requirements outlined by the Internal Committee of Medical Journal Editors. The study team anticipate preparing a “how to” guide for developing and implementing the DOAC Dashboard in health systems using the Epic® EHR. This guide will be made publicly available through appropriate requests to the MAQI^2^ organization.

### Anticipated challenges

While unlikely, it is possible that the VHA stakeholders generally dislike using the DOAC Dashboard and recommend against implementation in the MAQI^2^ centers. However, initial feedback from multiple VHA providers demonstrated a very positive reception and continued, growing use of the tool. Also, initial quality improvement work within MAQI^2^ has demonstrated that 50% of contacted providers end up changing the drug or dose when alerted [33]. Single-center data from VHA sites suggest that the DOAC Dashboard’s use increases pharmacist efficiency and results in more dosing changes than usual care.

Additionally, the COVID-19 pandemic has changed the way clinical care and clinical research are conducted in the USA. While the VHA Dashboard evaluation will include data from the pre-COVID-19 era, implementation in the MAQI^2^ centers will have to adapt for COVID-19 changes. These could include restricted staff resource to dedicate to the DOAC Dashboard and the need to access the dashboard while working remotely. From a clinical research perspective, data analysis can be performed using remote and secure virtual private networks and interviews can be conducted using videoconferencing or teleconferencing systems.

Lastly, while it is possible that some MAQI^2^ sites may have difficulty implementing the tool, we already have a working prototype developed within the Epic EHR at one center (University of Michigan). This demonstrates our ability to develop and implement such a tool and will provide valuable experience when implementing at other centers. We also have enthusiastic support from each of the health systems’ anticoagulation clinic leadership and a commitment to providing staff resources to implement and use the DOAC Dashboard for all patients within their health system.

## Discussion

Use of DOAC medications is growing rapidly both within the USA and globally. Given the increasing number of indications for anticoagulation and complexities around treatment, implementation of tools and processes to ensure safe use of these medications is critical. This study will accomplish three main goals: (1) evaluate the effectiveness of an EHR-based population health tool for medication management, (2) compare key determinants of implementation success within and outside of highly integrated health systems (e.g., VHA) to select implementation strategies, and (3) compare anticipated and experienced determinants of implementation success. These findings will help to inform future EHR-based implementation efforts in a wide variety of health care settings.

## Supplementary information

**Additional file 1.** VHA DOAC Dashboard Screenshots. ICD Codes for Adverse Clinical Events. DOAC Dashboard Barriers and Facilitators Exploratory Research, Discussion Guide for Anticoagulation Clinic (PharmD, RN)

## Data Availability

n/a

## Abbreviations

AF: Atrial fibrillation
CDW: Corporate data warehouse
CFIR: Consolidated Framework for Implementation Research
DOAC: Direct oral anticoagulant
EHR: Electronic health record
FDA: Food and Drug Administration
MAQI^2^: Michigan Anticoagulation Quality Improvement Initiative
PBM: Pharmacy Benefits Management
RE-AIM: Reach, Effectiveness, Adoption, Implementation, Maintenance
TAM: Technology Acceptance Model
VHA: Veterans Health Administration
VTE: Venous thromboembolism
