# Supplementary material for: Applying population health approaches to improve safe anticoagulant use in the outpatient setting: the DOAC Dashboard multi-cohort implementation evaluation study protocol
Source: Implement Sci. 2020 Sep 21;15:83. doi: 10.1186/s13012-020-01044-5 (PMC7504868; doi:10.1186/s13012-020-01044-5)
Supplement: Supplementary file 1 — Additional file 1. VHA DOAC Dashboard Screenshots. ICD Codes for Adverse Clinical Events. DOAC Dashboard Barriers and Facilitators Exploratory Research, Discussion Guide for Anticoagulation Clinic (PharmD, RN) [file 13012_2020_1044_MOESM1_ESM.docx]

**Online Appendix**

**VHA DOAC Dashboard Screenshots**


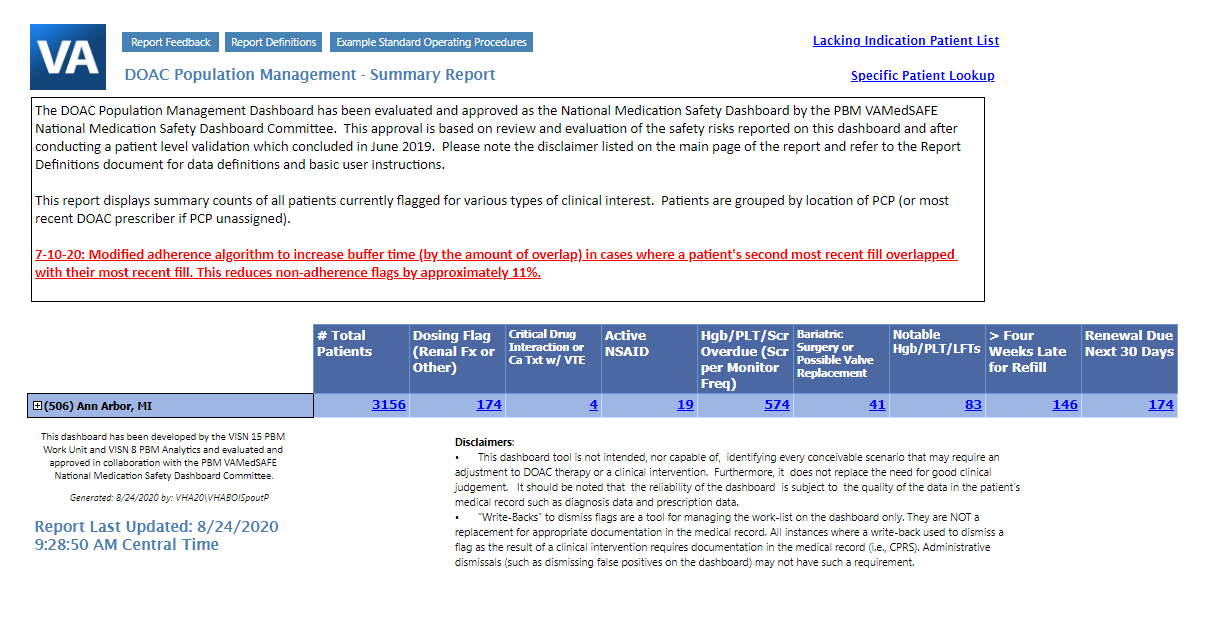

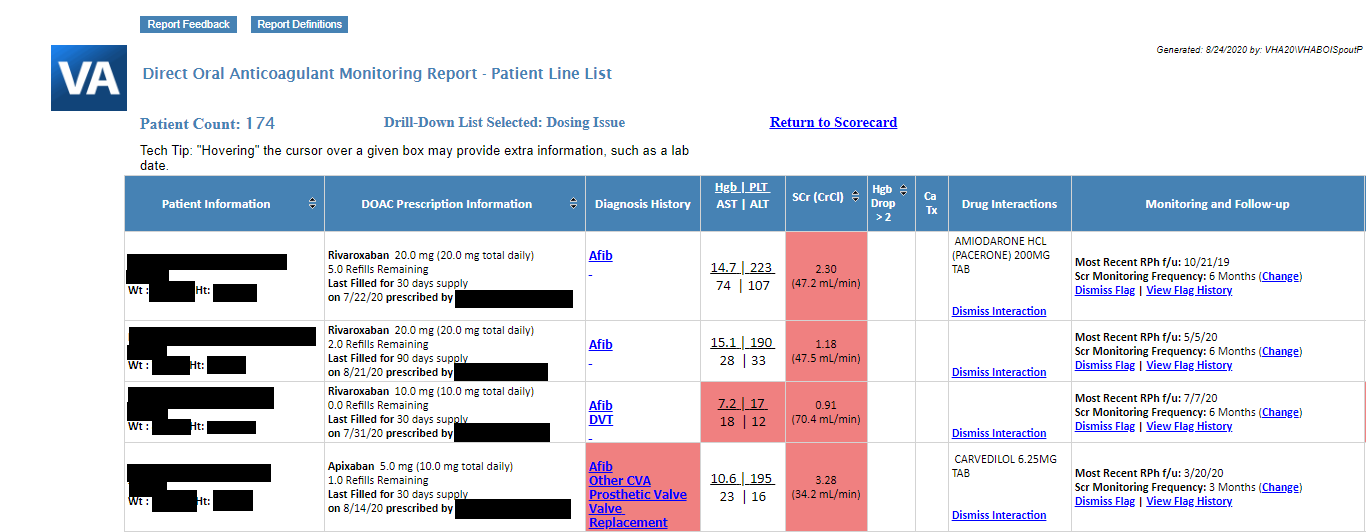


**ICD Codes for Adverse Clinical Events**

| **Adverse Event** | **ICD-9** | **ICD-10** |
| --- | --- | --- |
| **Stroke** | 433.01, 433.11, 433.21, 433.31, 433.91, 434.x, 435.x, v12.54 | I63, I64, G45 |
| **Venous Thromboembolism** | 453.4x, 453.5x, 451.1x, 451.8x, 451.9, V12.55 | I82.4x, I82.5x, I27.82, Z86.711, I26.x |
| **Bleeding** (using approach from Jasuja 2013) | 430, 431, 432.1, 432.9, 658.81, 719.16, 455.2, 455.5, 455.8, 459.0, 530.7, 535.01, 535.41, 535.51, 537.83, 562.12, 562.13, 569.3, 569.85, 578.0,578.1, 578.9, 596.7, 599.7, 782.7, 784.7, 786.3 | I97.620, K91.84x, H95.42, E89.811, H59.32x, N99.82x, H95.41, M96.83x, G97.5x, J95.83x, L76.2x, D78.2x |

**DOAC Dashboard Barriers and Facilitators Exploratory Research**

**Discussion Guide for Anticoagulation Clinic (PharmD, RN)**

- 30-45-minute interviews with non-, medium-, and high-use sites VA sites

Objective: Understand known and perceived barriers and facilitators to use of DOAC Dashboard tool within the respondent’s own facility

1. Identify human facilitators which have contributed or would contribute to adoption of or resistance to the DOAC Dashboard on site
2. Identify communication strategies that influenced DOAC Dashboard adoption and use
3. Identify the logistical barriers and facilitators that can drive adoption of the DOAC Dashboard
4. Identify the forms of infrastructure that benefit or limit adoption and use of the DOAC Dashboard

*NOTE: This discussion guide acts as an outline for the conversation between the respondent and the researcher. Researchers have made efforts to craft all probes that may be needed, but the interviewer may ask additional questions to meet the overarching and specific research objectives. Language and question order may be modified for the researcher’s or respondent’s comfort and needs and to improve the flow of the questions and conversation.*

| **Group** | **Questions and Probes** |
| --- | --- |
| All | - 1. Interviewer introduction, consent, and overall study objectives: *My name is _____, and I’m a researcher working with the VA in Ann Arbor. This interview is a part of a larger research study we’re conducting with people who are involved in the treatment and care of patients taking anticoagulants, specifically DOACs. We are trying to understand how work flows in caring for these patients and how technological tools that evaluates patients on DOACs does or could fit into that work flow. There are no right or wrong answers, and all of your responses and this recording are completely confidential and will be stored on a secure server without any identifying information about you or your employer or location.* *You may ask me to turn the recorder off at any point.* |

| **Group** | **Questions and Probes** |
| --- | --- |
| All | **Introduction and Warm-up**   - 1. Professional background      1. Practice setting, job title, years in this kind of setting, years in current role      2. How many patients on anticoagulant therapy do you care for/review per month? In what capacity?      3. Can you describe for me the type of anticoagulation care your clinic provides?         1. Inpatient vs. outpatient?         2. What types of diagnoses (AFib, VTE, post-operative, other)?         3. Which drugs do you support (warfarin, DOAC, other)?  1. How are patients referred to the anticoagulation clinic?    - - 1. Do ALL anticoagulated patients get referred/followed or only select patients?        2. Which providers (e.g. PCP, cardiologist, surgeon) refer patients to the anticoagulation clinic?        3. Which types of providers (e.g. specialties, location) do you support during your care of anticoagulated patients? 2. What types of services do you provide within your anticoagulation clinic?    - - 1. Do you provide one-time review? Ongoing care?        2. Do you help select best drug/dose? Peri-op care? Patient education? Other?        3. How much time, on average, do you spend reviewing the chart and interacting with a new anticoagulation patient? A follow-up?        4. What questions do patients normally ask you during your interactions? |
| All | **DOAC Dosing and Medication Errors: Current State**   1. In general, what barriers do you experience getting your patients optimally treated with DOACs?    1. Probe: Prescriber-related; patient-related; system-related 2. In general, within your hospital or clinic, what are the most common DOAC prescribing errors/problems? Who usually identifies these problems? (if necessary: without the DOAC Dashboard tool)? What is the process for handling these errors/problems?    - 1. What tools do you have available to you to make sure that your patients on DOACs are appropriately managed?      2. What role does the anticoagulation clinic have in identifying and managing patients on DOACs (vs. other providers, e.g., cardiology/PCP)      3. Who are the people who are influential in anticoagulation care within your health center? |
| All | - 1. In what ways does the medication error identification process break down? What barriers do you have with the current flow? Which parts take the longest time?   2. In what ways has it been successful? |
| All | - 1. How have these processes changed in recent years at your site?      1. Probes:         1. Use of Dashboard         2. Changes in anticoagulation clinic structure with rise of DOACs. |
| VA non-user | 1. Present respondent with DOAC DASHBOARD description and images: *This is a description of a tool that is / can be built into the electronic health record of your facility to evaluate patient records and flag any patients who are taking a DOAC and have a contraindication or dosing error. Feel free to write on this. What catches your attention? What do you have questions about?*     - 1. What are your initial impressions? / Talk me through your notes.      2. Seek to understand the following:         1. How do you see yourself using this tool?         2. Do you use something similar now?         3. What benefits do you see to this tool in your current workflow?         4. Drawbacks?      3. What questions would you have for the developer?      4. Who in your workgroup would be the biggest champion of a tool like this? The biggest hurdle?      5. How would you get your colleagues to use this tool?      6. Can you tell me about a time when a tool or new process was rolled out that you think it went really well?      7. What about a time where it could have gone better? |

| **Group** | **Questions and Probes** |
| --- | --- |
| VA non-user | 1. Revisit barriers, resources, timing, unmet needs: 2. Which of your prior barriers / process does this tool address? 3. What do you think would be more complicated or difficult if this tool were adopted in your center? 4. What information about this tool would be most important to the people deciding whether to use it? 5. What other features or benefits could a tool like this have to address any barriers that patients in your setting have to safe and effective DOAC use? |
| VA Dashboard Users | 1. Tell me about the DOAC tool you use for the patients in your facility.    - - 1. Tell me about how you first heard about this tool?        2. Tell me about how you first learned to use this tool?        3. How long would you say it was before you were comfortable using this tool?        4. When did your team start using it? When did you start using it? 2. Who in your team brought it on board for use? (title, role)    - - 1. How did that person explain the tool? Were they a champion for use of the tool or just a messenger?        2. What champions were influential in getting the DOAC dashboard used? Why were they influential?        3. What was the process like when you first started using the DOAC Dashboard? (growing pains, integration into workflow, dealing with initial backlog of patients/flags) 3. What has the overall impact of this tool been in your work and patient care? 4. Probe on time spent, communication with colleagues and patients, impact on patients directly 5. What are the benefits you see in using this? 6. Drawbacks? 7. What has limited your use of this tool in your current setting? 8. Process requirements, colleagues, red tape 9. What has expanded your use of the tool? 10. Can you give me an example where the DOAC Dashboard has been particularly useful? 11. An example where it created unnecessary problem or headache? 12. What other patient management tools are you using in your day to day work? 13. How well does the DOAC Dashboard fit into workflow with the other Dashboards or tools you use? 14. How would you improve this tool? 15. What features could be added to this tool to further benefit patient care? 16. What features could be removed? |
| All | 1. What else can the VA do to support safe DOAC use? 2. Which other staff or providers should we contact to learn more about how the DOAC Dashboard was/could be implemented in your health center? 3. Anything else you would like to share about your experiences? Any other questions we should be asking? |
